# Eccentric magnetic microcapsules for MRI-guided local administration and pH-regulated drug release†

**DOI:** 10.1039/c8ra08501c

**Published:** 2018-12-17

**Authors:** Jingxian Huang, Wenwei Huang, Yin Chen, Yu Shrike Zhang, Jinshuang Zhong, Yan Li, Jianhua Zhou

**Affiliations:** School of Biomedical Engineering, The First Affiliated Hospital, Sun Yat-sen University Guangzhou 510006 China chenyin8@mail.sysu.edu.cn zhoujh33@mail.sysu.edu.cn +86 20 39387890 +86 20 39387890; Division of Engineering in Medicine, Department of Medicine, Brigham and Women's Hospital, Harvard Medical School 65 Landsdowne Street Cambridge Massachusetts 02139 USA; Imaging Department, Sun Yat-sen University Cancer Center, Sun Yat-sen University Guangzhou 510080 China

## Abstract

In this work, we present a novel class of uniform eccentric magnetic microcapsules based on polydimethylsiloxane (PDMS) for magnetic resonance imaging (MRI)-guided local injection and pH-regulated drug release. The microcapsules contained magnetic nanoparticles in their PDMS shells, which allowed them to be easily tracked by MRI during administration. Besides, they showed pH-dependent drug release profiles due to dissolution of the embedded magnetic nanoparticles in acidic solutions. Moreover, by tuning the mass fraction of the magnetic nanoparticles, we could further regulate the release rate of drug molecules from them. As a demonstration, we investigated the delivery of cis-platinum using the microcapsules through an *in vitro* cell test, which confirmed the pH-controlled release of the drug in phantom tissues. Our study suggests that this type of eccentric magnetic microcapsules could be simultaneously employed as a potential imaging contrast and a smart drug delivery system, holding great potential for guided local therapy.

## Abstract 2

Uniform eccentric magnetic microcapsules for magnetic resonance imaging-guided local administration and pH-regulated drug release were developed.

## Graphical abstract

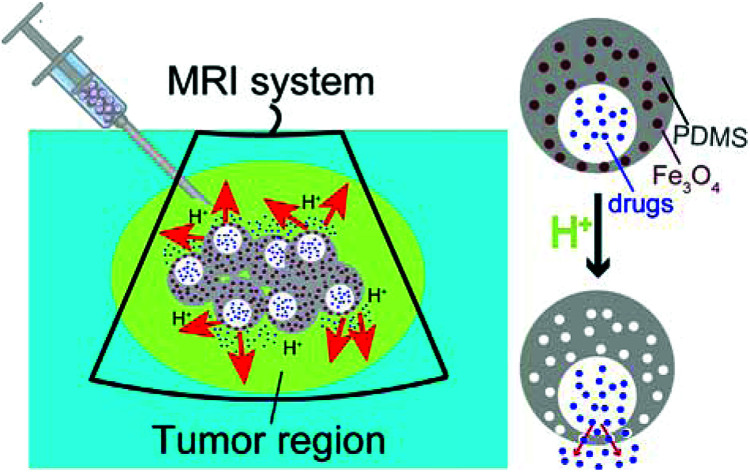

## 1. Introduction

In the past few decades, considerable attention has been paid to the design of multi-functional microcarriers that are capable of controlled drug release and visualization during administration. Improving the efficacy of drugs at lesion sites and minimizing their systemic side effects are the main goals for this type of drug delivery systems compared to conventional ones.^1–6^ In particular, local injection of a controlled drug delivery system has been widely adopted in scientific research and in clinical trials such as for the treatment of cancer.^7–15^ However, due to the complex anatomy of the human body, implementation of a precise local injection is not always feasible.^16^ Therefore, it is highly necessary to design a type of microcarriers that can be visualized during injection to minimize unnecessary organic injury and unwanted side effects. So far, ultrasound imaging (US), computed tomography (CT), and magnetic resonance imaging (MRI) are imaging modalities that are extensively utilized in clinical practice. Although the literature has reported the local injection of drug carriers guided by US or CT,^16–18^ the low resolution of US and the potentially risky exposure to ionizing radiation from CT remain challenges. As an alternative, MRI has become increasingly popular for this purpose in recent years thanks to its safety and high quality of clinical imaging.

Besides the need for visualization, an ideal drug delivery system should also be capable of controlled drug release. To date, various types of polymer microcarriers have been developed.^19–34^ In principle, they achieve controlled drug release through either external stimuli^19–27^ or internal stimuli.^28–34^ External stimuli can be ultrasonic stimulation,^19–21^ laser stimulation^22,23^ magnetic field,^24–26^ or mechanical strain^27^ while internal stimuli include temperature,^28–30^ pH,^31–33^ and enzymolysis.^34^ Internal stimuli can be exploited for realizing self-regulation of drug release, which is more intelligent than the way by external stimuli. Although MRI is now frequently utilized for guided local therapy,^35–38^ no study has reported any microcarrier system that combines MRI contrast and drug release regulated by pH, which varies dramatically between lesion sites (such as cancer) and normal tissues.

**Scheme 1:**
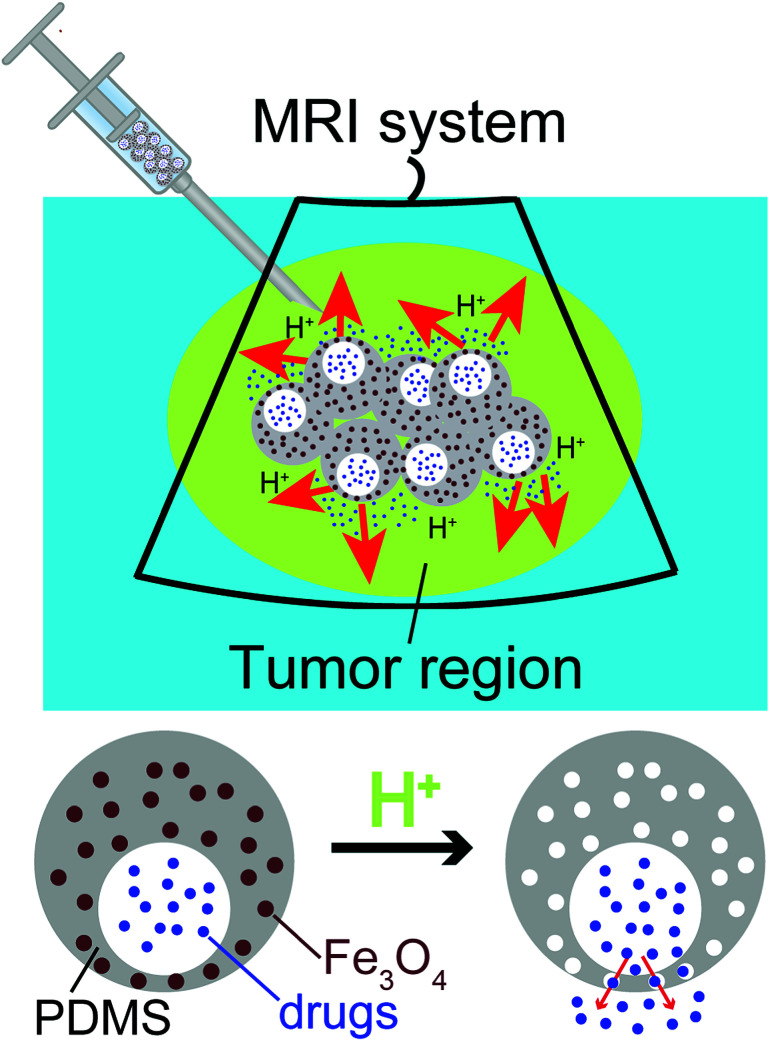
Diagram showing MRI-guided local administration of the eccentric magnetic microcapsules and pH-regulated drug release from them at the tumor region.

To fill this gap, we have developed a novel class of uniform eccentric magnetic microcapsules based on polydimethylsiloxane (PDMS) for MRI-guided local administration and pH-regulated drug release (Scheme 1). Using a three-phase microfluidic device,^39–41^ we obtained eccentric microcapsules containing magnetic nanoparticles within their PDMS shells. The embedded magnetic nanoparticles enabled easy visualization using an MRI scanner, thereby achieving accurate imaging-guided local injection. Because of the solubility of the magnetic nanoparticles in acidic solutions, they could also function as porogen in the PDMS shell and enhance drug release at lower pH values. In our study, we demonstrated pH-dependent release profiles of graphene quantum dots (GQDs) and sodium fluorescein as model drugs. Moreover, cis-platinum was loaded into the eccentric magnetic microcapsules and its release behavior and therapeutic efficacy on cancer cells in phantom tissues were investigated.

## 2. Experimental section

### 2.1 Chemicals and materials

PDMS prepolymer (Dow Corning, USA) and dichloromethane (DCM, Damao Reagent, China) were used as the main components for the preparation of microcapsules. Oil-soluble Fe_3_O_4_ magnetic nanoparticles (∼10 nm, modified by oleic acid)^42^ were fully dispersed in the PDMS prepolymer solution at varying mass fractions as needed. Polyvinyl alcohol (PVA, Sigma-Aldrich, USA) at a concentration of 2.0 wt% served as both the inner and outer water phases. GQDs (∼2.5 nm in diameter) are prepared according to Shi's work,^43^ sodium fluorescein (Aladdin, China) and cis-platinum (Aladdin, China) were used as model drugs and suspended/dissolved in the inner phase. Glass capillary tubes (two different sizes with inner/outer diameters of 0.70/1.23 and 0.45/0.65 mm, respectively) were purchased from Ace Glass (USA). Deionized (DI) water used in all experiments was acquired from a set of Millipore cartridges (Epure, Dubuque, IA, USA).

### 2.2 Preparation of the eccentric magnetic microcapsules

The microfluidic device was consisted of two polyvinyl chloride (PVC, Boyun, China) tubes (0.60/1.50 and 1.45/2.30 mm in inner/outer diameters, respectively), two glass capillaries, and a 30 G needle, which was fabricated according to our previous work with minor modifications (Fig. S1†).^44,45^ Briefly, it was assembled by inserting the needle and glass capillaries into PVC tubes, and then sealed with epoxy adhesive. The inner, middle, and outer phases were introduced using three individual syringe pumps (KD100, KD Scientific, USA). In this work, the flow rates for the inner, middle, and outer phases were maintained at 0.004, 0.03, and 1.5 mL min^−1^, respectively, to generate the eccentric microcapsules. The inner water droplets formed at the tip of the needle and flowed along the PVC tube into the small glass capillary tube. Subsequently, the water-in-oil droplets formed at the exit of the small glass capillary tube and finally flowed along the larger glass capillary tube into a 100 mL beaker containing the outer water phase. The model drug-containing PVA solution served as the inner water phase. The mixture of PDMS prepolymer and DCM (3 : 1 in mass ratio) supplemented with oil-soluble magnetic nanoparticles were used as the middle oil phase. PVA solution (2 wt%) was used as both the outer water phase and collection solution. The water-in-oil droplet suspensions were kept at 60 °C for 30 min to remove DCM, and then at 90 °C for 30 min to solidify the droplets. For static solidification, the water-in-oil droplets were maintained static to allow the inner water phase to float to the top due to the lower density. Finally, we harvested the uniform eccentric microcapsules with the magnetic nanoparticles evenly dispersed in their PDMS shells.

### 2.3 Characterization of the eccentric magnetic microcapsules

An inverted fluorescence microscope (Ti-U, Nikon, JPN) with a CCD camera (Digital Sight DS-Fi2, Nikon, JPN), a stereoscope (SZ760-DM601, Optec, China) and a scanning electron microscope (SEM, JSM-6010LA, JEOL, JPN) were used to characterize the morphology and structure of the eccentric magnetic microcapsules. The samples for SEM were prepared by slicing the eccentric magnetic microcapsules in halves along the vertical plane of the thin wall and then sputter-coating the sections of them with gold. Their average diameters and the standard deviations were calculated by measuring over 50 microcapsules randomly selected from the optical micrographs of each group. SEM was also applied for the elemental analysis of iron distribution in the microcapsules. An optical coherence tomography (OCT) scanner (HSO-2000, Teksqray, China) and an MRI scanner (Trio Tim 3.0T, Siemens, GER) were used to visualize those injected into the phantom agarose hydrogels (Aladdin, China).

### 2.4 UV/vis absorption spectrum measurement

An ultraviolet-visible (UV/vis) spectrophotometer (UD730, Beckman, USA) was used to measure the release profiles of model drugs from the microcapsules. Each drug was loaded into the microcapsules by adding it into the PVA solution of the inner water phase (5 mg mL^−1^ for GQDs, 0.25 mg mL^−1^ for sodium fluorescein, or 0.45 mg mL^−1^ for cis-platinum). After solidification, the microcapsules (0.5 g for each sample) were dispersed in 3 mL of phosphate-buffered saline (PBS) at the pH of 5.5, 6.0, 6.5, 7.0, or 7.5. The UV/vis absorption spectra of the solutions were determined every 5 min for the first 15 min and every 15 min afterwards. The intensity at the maximum absorption wavelength (359 nm for GQDs, 491 nm for sodium fluorescein, or 194 nm for cis-platinum) was recorded as a function of exposure time.

### 2.5 *In vitro* cell test

HeLa cells transfected with green fluorescent protein were used in this study. They were cultured in Dulbecco's modified Eagle medium (DMEM, high-glucose) supplemented with 10% fetal bovine serum (FBS) and 1% penicillin/streptomycin (P/S), and incubated at 37 °C with 5% CO_2_. The medium was replaced every 2 days. At 80–90% confluence, the cells were washed twice with PBS (pH 7.4), detached with 0.25% trypsin–EDTA solution, and then resuspended in 0.1 g mL^−1^ gelatin (Aladdin, China) solution (dissolved in PBS), of which the pH varied. After encapsulation of HeLa cells, the gelatin solutions were transferred into a 24-well plate (1.0 mL for each well). When the solidification completed, three microcapsules (containing cis-platinum) were injected into each gelatin hydrogel. Fluorescence photographs of the samples (underneath the microcapsules) were taken every 30 min with an inverted fluorescence microscope (IX71, Olympus, GER).

### 2.6 Statistical study

Experimental results were presented as mean ± standard deviation and statistical significance was determined by Student's *t*-test.

## 3. Results and discussions

### 3.1 Morphology and iron distribution of the eccentric magnetic microcapsules

**Fig. 1:**
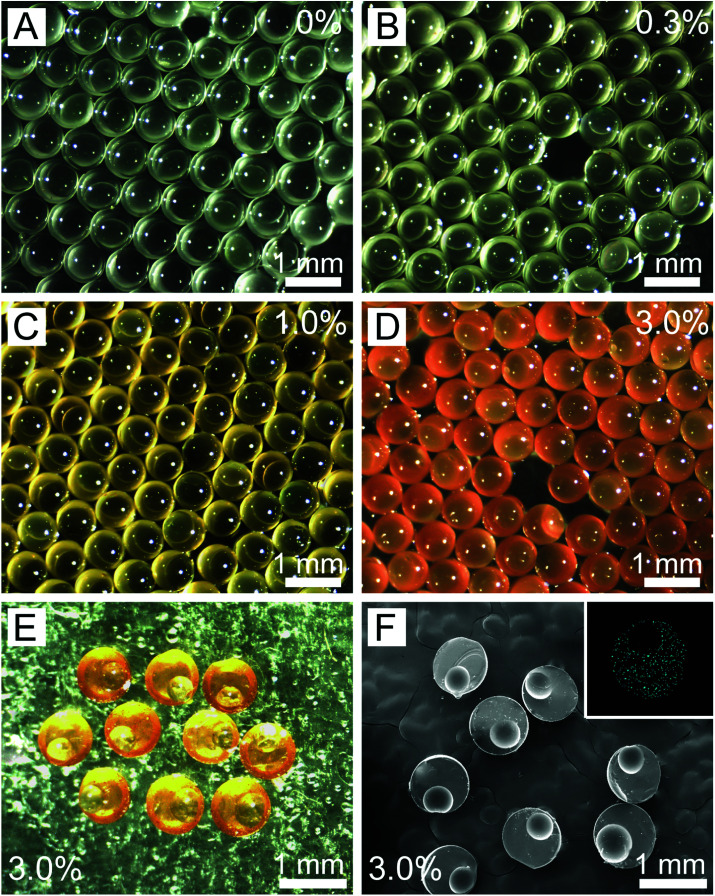
Morphology and structure of the eccentric microcapsules with varying mass fractions of the magnetic nanoparticles. (A–D) Optical images of the microcapsules with varying mass fractions of the magnetic nanoparticles in the PDMS shells from 0% (A) to 0.3% (B), 1.0% (C), and 3.0% (D). (E) Optical image showing the cross-sections of the eccentric magnetic microcapsules. (F) SEM images of the sliced eccentric magnetic microcapsules showing the cross-sections and (inset) the elemental analysis of iron distribution in them.

Using our method, we successfully fabricated the eccentric microcapsules with non-uniform wall thickness (Fig. 1). The main component of our eccentric magnetic microcapsules is PDMS, which is a biocompatible material widely used as oral pharmaceutical excipient.^46,47^ Magnetite or Fe_3_O_4_, is the most-studied spinel ferrite.^48^ Fe_3_O_4_ nanoparticles are considered to be biologically and chemically inert, which are particularly useful for drug targeting and magnetic resonance imaging.^49^ The oil-soluble magnetic nanoparticles were directly loaded into the middle oil phase (PDMS prepolymer solution). Fig. 1A–D shows images of the uniform microcapsules with varying mass fractions of the magnetic nanoparticles in PDMS. It could be observed that they changed from colorless to orange with the increase in the content of the magnetic nanoparticles. These microcapsules were all eccentric as a result of the static solidification, during which the inner aqueous cores floated to the top of the droplets due to the lower density. However, these aqueous cores could not penetrate the shells and escape due to the low surface energy of PDMS,^46,47^ eventually leading to the formation of eccentric microcapsules with a very thin layer of PDMS on one side.

We further inspected the internal structures of the eccentric magnetic microcapsules by slicing and then examining them under an SEM. SEM images indicated that all microcapsules were uniform in size (Fig. 1F), with the average diameters of themselves and their internal spherical cores being 808.8 ± 3.5 μm and 482.2 ± 2.0 μm, respectively. Moreover, the thinnest part of the PDMS shell was only about 8.1 ± 0.3 μm, which could possibly function as the “exit” for drugs. Elemental analysis revealed that iron distribution was homogenous in PDMS (Fig. 1F, inset).

In addition, we also observed the three-dimensional (3D) structures of the magnetic microcapsules under an OCT scanner. Clear images were obtained because of the enhanced scattering by the magnetic nanoparticles in PDMS. Fig. S2A–C† shows the cross-sections of a microcapsule in three orthogonal planes. By taking a series of images, the 3D reconstructed image was generated, clearly displaying the eccentric structure of the microcapsule (Fig. S2D†).

### 3.2 MRI-guided local administration of the microcapsules

As a proof of concept for MRI-guided local administration, the eccentric magnetic microcapsules (containing different mass fractions of magnetic nanoparticles) were injected into a hydrogel slab while being observed under an MRI scanner. The hydrogel was used as a phantom tissue here (Fig. S3†). As shown in Fig S4A,† the signal of the microcapsules was very strong when the mass fraction of the magnetic nanoparticles in PDMS was 3.0%. However, it declined dramatically when the mass fraction was reduced to 1.0% (Fig. S4B†). As expected, the microcapsules without the supplement of the magnetic nanoparticles could hardly be distinguished from the background of MRI (Fig. S4C and D†), which fully demonstrated the role of the magnetic nanoparticles as an MRI contrast agent.

**Fig. 2:**
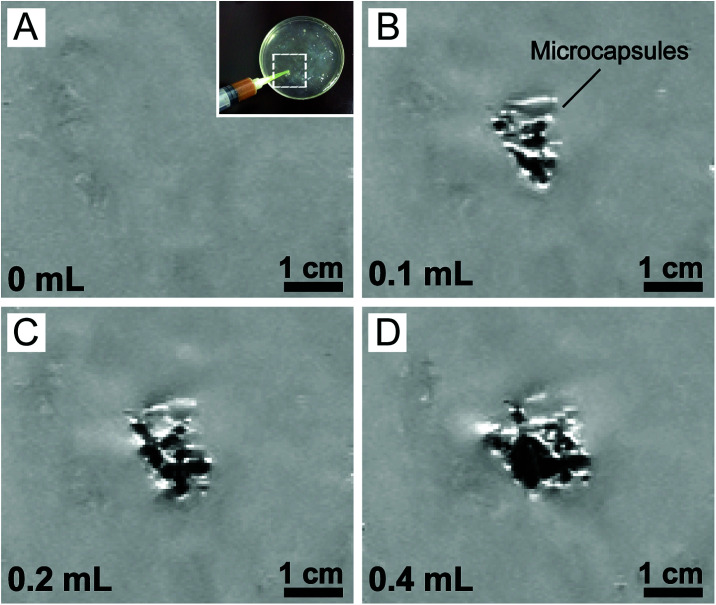
MRI images of varying amounts of the eccentric magnetic microcapsules (containing 3.0% magnetic nanoparticles) in hydrogel phantom tissues. (A) MRI image at the designated region (inset) of a blank hydrogel slab (without microcapsules). (B–D) MRI images of the hydrogel slabs injected with increasing amounts of the microcapsules at 0.1 mL, 0.2 mL, and 0.4 mL, respectively. (0.1 mL microcapsules ≈ 150 microcapsules.)

In addition, we also tuned the amount of the magnetic microcapsules (containing 3.0% magnetic nanoparticles) for injection into the hydrogel tissue phantom. MRI images showed that the region of interest (ROI) enlarged accordingly with the increase in the amount of the magnetic microcapsules (Fig. 2), corroborating their successful visualization by MRI. However, owing to the low resolution of our MRI scanner, individual microcapsules could not be identified. Considering the similarities between hydrogels and real soft tissues,^50,51^ our results suggested that the eccentric magnetic microcapsules should also be useful for guided local therapy in the human body.

### 3.3 pH-dependent release of model drugs from the microcapsules

**Fig. 3:**
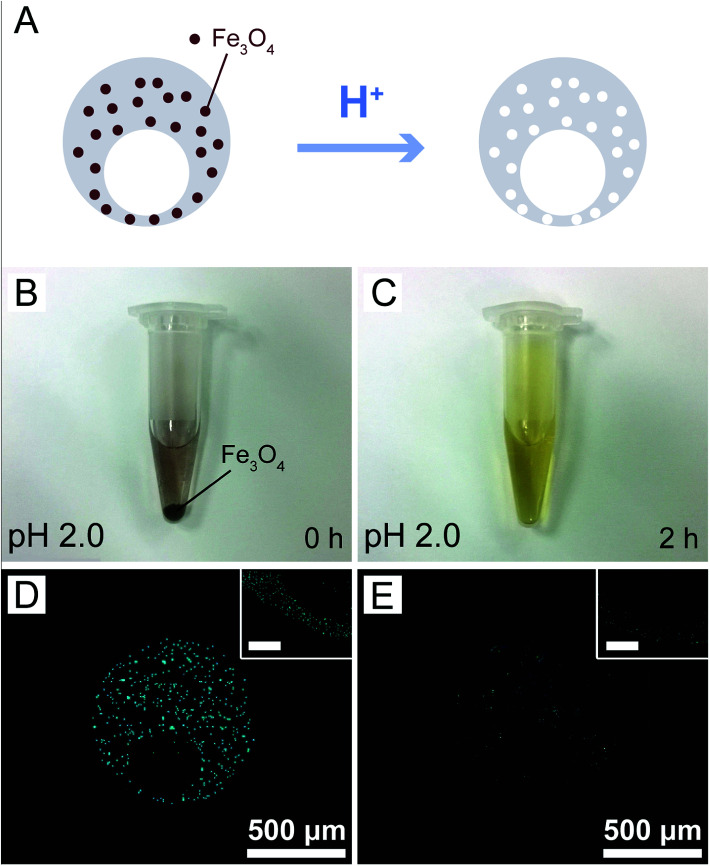
Dissolution of Fe_3_O_4_ magnetic nanoparticles in acidic solutions. (A) Schematic diagram for the dissolution of the magnetic nanoparticles (dispersed in the microcapsules) in an acidic condition. (B and C) Photographs showing the dissolution (pH 2.0, 2 h) of the nanoparticles deposited at the bottom of a plastic tube. (D and E) Elemental analysis of iron distribution in the microcapsules before (D) and after (E) acidic treatment (pH 6.0, 2 h). The insets (scale bars: 10 μm) are higher-magnification images of the thinnest part of the PDMS shells.

The magnetic nanoparticles used in this study had been modified by oleic acid and were fully dispersed in the PDMS prepolymer mixture during microcapsule fabrication. More importantly, they could be dissolved by acids, leading to the formation of nanopores in the PDMS shells of the microcapsules as illustrated in Fig. 3A. Before investigation on the release profiles of model drugs from the microcapsules, we investigated the dissolution of the magnetic nanoparticles in acidic solutions. Although a strong acidic solution (pH 2.0) was required to rapidly (2 h) dissolve the nanoparticles deposited at the bottom of a plastic tube (Fig. 3B and C), those uniformly dispersed in PDMS could be easily removed under a very weak acidic condition (pH 6.0) within the same period of time, as indicated by the elemental analysis of iron (Fig. 3D and E). Indeed, the microcapsules embedded with 3.0% magnetic nanoparticles showed a much higher release rate of the loaded sodium fluorescein than those without the nanoparticles at pH 6.0 (Fig. S5†). Such results suggested that our magnetic microcapsules were pH-sensitive due to the dissolution of the embedded magnetic nanoparticles in an acidic solution and thus they could be exploited for controlled drug release.

**Fig. 4:**
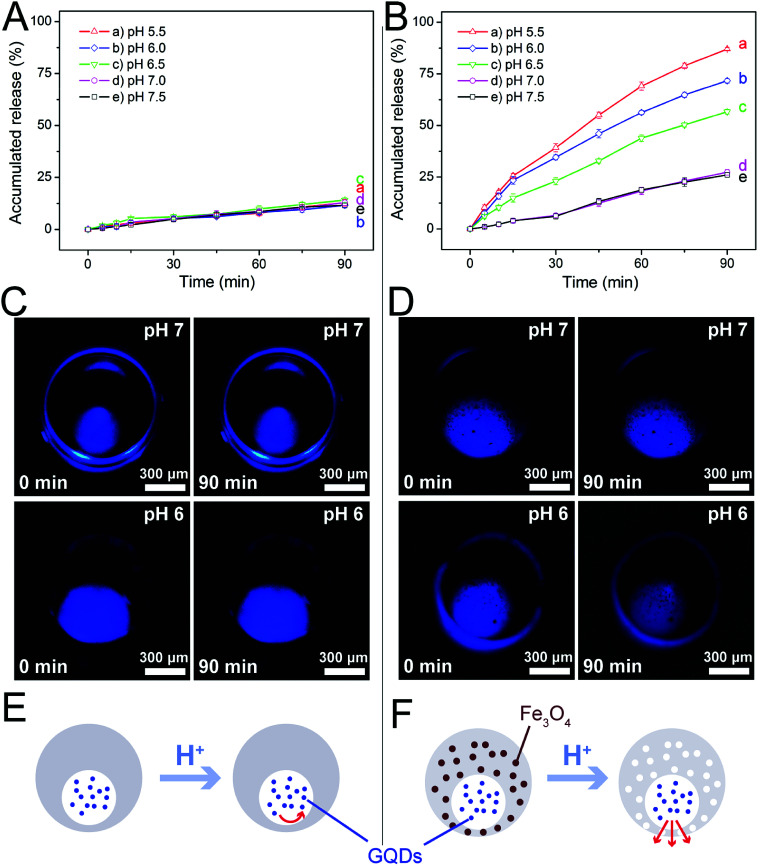
Release of GQDs (∼2.5 nm) from the eccentric magnetic microcapsules. (A and B) Release profiles of GQDs from the microcapsules without (A) or with 3.0% magnetic nanoparticles (B) under varying pH values (5.5 to 7.5). (C and D) The fluorescence images of GQD-loaded eccentric microcapsules without magnetic nanoparticles (C) or containing 3.0% magnetic nanoparticles (D) under two typical pH conditions for a period of time. (E and F) Schematic illustrations for the different release behaviors of GQDs from microcapsules without (E) or with (F) magnetic nanoparticles.

Encouraged by these results, we studied the influence of pH on drug release from our magnetic microcapsules, where GQDs (∼2.5 nm in diameter) were applied as the first model drug. Microcapsules without magnetic nanoparticles were used as the control group. The release profiles of GQDs from them at varying pH conditions (5.5 to 7.5) are shown in Fig. 4. It was observed that the release of GQDs was barely affected by pH without the presence of magnetic nanoparticles (Fig. 4A). In fact, the fluorescence images in Fig. 4C also revealed little reduction in fluorescence intensity in 90 min for GQDs loaded in these control microcapsules at two pH conditions (pH 6.0 and pH 7.0), suggesting that GQDs were hardly released. However, when the magnetic nanoparticles were included in the microcapsules, the release profile of GQDs was evidently affected by pH, with higher release rates observed at lower pH values (Fig. 4B), due the formation of nanopores after the nanoparticles had been dissolved by the acid. Fig. 4D shows the fluorescence images of the GQD-loaded magnetic microcapsules (3.0% magnetic nanoparticles) at two pH conditions (pH 6.0 and pH 7.0). The prominent reduction in fluorescence intensity of GQDs suggested that some of them had been released from the magnetic microcapsules into the surrounding medium, which corroborated the results in Fig. 4B. By exploiting the degradation of the magnetic nanoparticles in acidic conditions, we successfully prepared magnetic microcapsules that could simultaneously achieve visualization by MRI and pH-regulated release of the loaded GQDs from them.

These results suggested that we could use different pH conditions to regulate the release rate of a drug. PDMS is a water-permeable and elastomeric material allowing for tunable pore size for drug release.^44,47^ However, the native pore size of PDMS is so small that the permeability is not sufficient for the loaded drug molecules to diffuse through the PDMS shell (Fig. 4E). In comparison, after blending with the magnetic nanoparticles, our microcapsules became pH-sensitive. Under acidic conditions, the dissolved magnetic nanoparticles could leave behind large pores in the PDMS shell, thereby improving its permeability to facilitate the release of the drug molecules from the microcapsule (Fig. 4F).

**Fig. 5:**
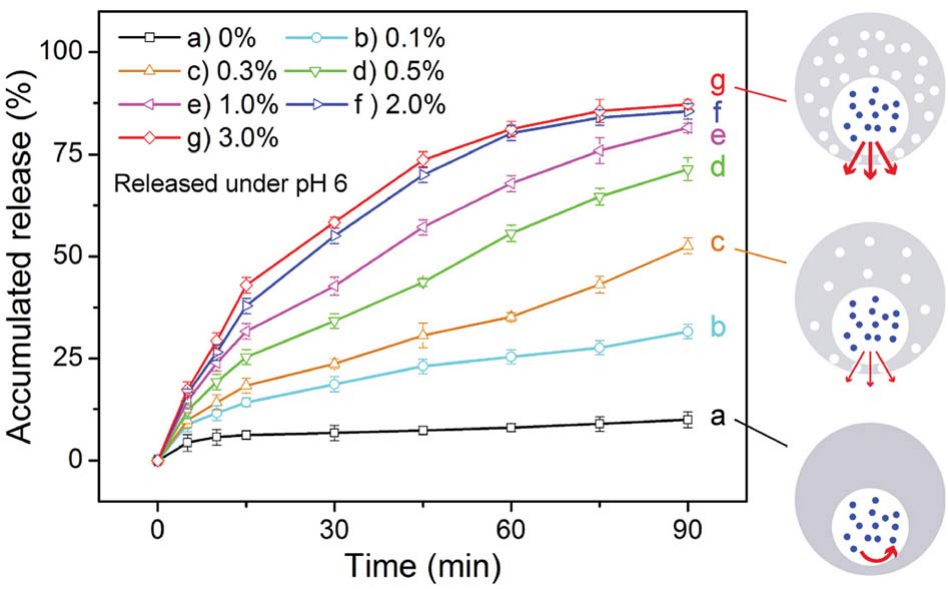
Effect of the mass fractions of the magnetic nanoparticles on the release rate of sodium fluorescein from the microcapsules (pH 6). The mass fractions of the magnetic nanoparticles range from 0% to 0.1%, 0.3%, 0.5%, 1.0%, 2.0%, and 3.0%.

Except for the influence of pH, we also studied the effect of the concentration of the magnetic nanoparticles on the release rate of a drug from the eccentric magnetic microcapsules. For this we used sodium fluorescein as the model drug, which was loaded into a series of microcapsules with varying mass fractions of the nanoparticles (0% to 3.0%). The pH of the incubation medium was set to 6.0 to induce drug release. As shown in Fig. 5, sodium fluorescein was released at different rates from the microcapsules with varying mass fractions of the nanoparticles. In general, a higher content of the magnetic nanoparticles facilitates a higher release rate of sodium fluorescein. This could be explained by the fact that as the more nanoparticles existing the PDMS shell, the more nanopores would be formed. Besides, a higher content of the magnetic nanoparticles will also provide a higher magnetic property (Fig. S6†), which could make the EMMs more sensitive to the magnetic field to realize more efficient magnetic imaging and potential electromagnetic effect. Such results demonstrated that the drug release process could also be regulated by the amount of magnetic nanoparticles, providing more flexibility for our microcapsules serving as vehicles for controlled drug delivery.

Furthermore, we compared the release profiles between GQDs and sodium fluorescein under two common pH conditions (pH 6.0 and pH 7.0). As shown in Fig. S7,† both model drugs presented high release rates under pH 6.0 and low release rates under pH 7.0. However, the release rate of GQDs at a given pH value was lower than that of sodium fluorescein on account of their larger size.

### 3.4 *In vitro* cell test of the microcapsules for pH-regulated drug release

**Fig. 6:**
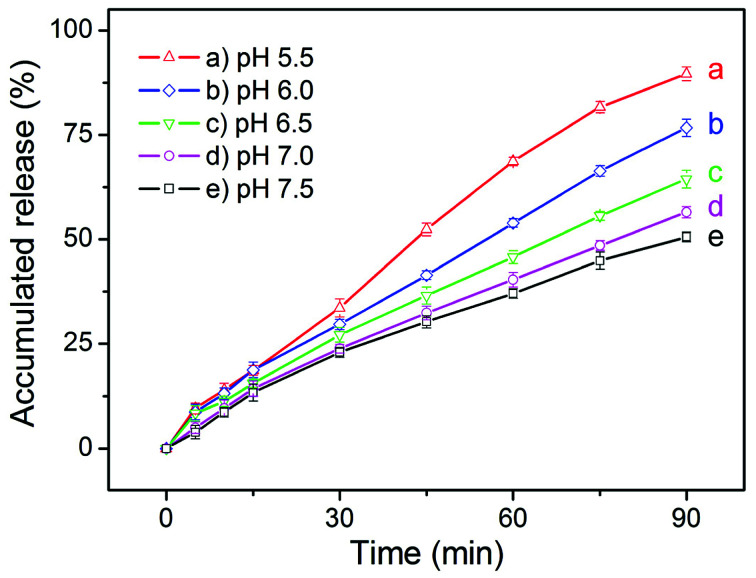
The pH-dependent release profiles of cis-platinum from the eccentric magnetic microcapsules (3.0% magnetic nanoparticles).

To evaluate the feasibility of our eccentric magnetic microcapsules for MRI-guided local administration and pH-controlled drug release, we carried out an *in vitro* cell test. First, we determined the release profiles of cis-platinum (an anti-cancer drug) under various pH conditions. As shown in Fig. 6, the general trend for the release profiles of cis-platinum was similar to that of GQDs, *i.e.* higher release rates were observed at lower pH values. However, the release rate of it (approaching 50% in 90 min) was much higher than that of GQDs (less than 25% in 90 min) when the pH was 7.0 or 7.5, which was likely caused by the much smaller size of the anti-cancer drug.

Subsequently, we evaluated the biocompatibility of our microcarriers *in vitro* (without any drugs) based on the viability of GFP-HeLa cells. We co-cultured the cells with blank eccentric magnetic microcapsules at varying mass concentrations, after which MTT assay was performed to determine their viabilities. As shown in Fig. S8,† although cell viability slightly decreased with the increase of blank microcapsules, more than 80% of the cells were still alive even after incubation with 15 mg mL^−1^ blank microcapsules for 5 days, confirming the good biocompatibility of them. To exclude the influence of weak acidic environment itself on GFP-HeLa cells, we incubated them without microcapsules for 90 min at pH 6 and pH 7, respectively. Our result (Fig. S9†) suggested the cell viability at pH 6 was not significantly different from that at pH 7. This result demonstrated that the weak acidic environment (pH 6) would not impair the viability of HeLa cells during the experiment.

**Fig. 7:**
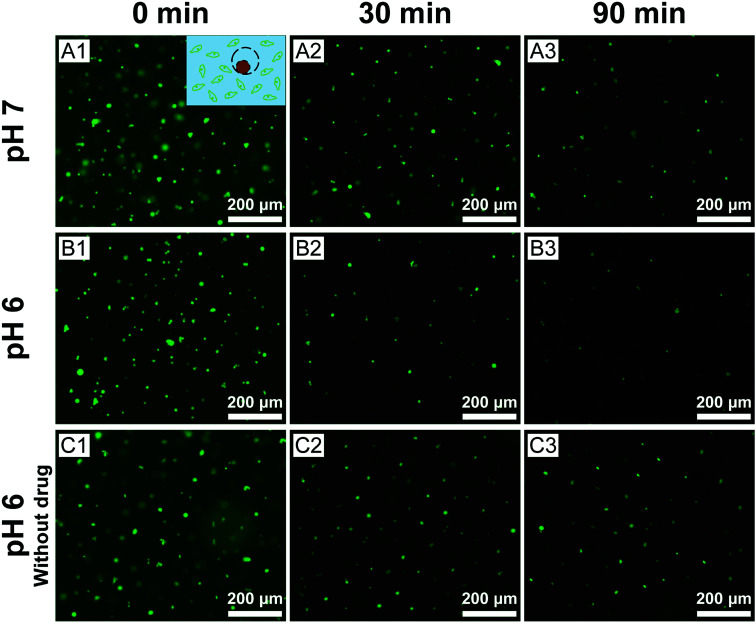
Fluorescence images of the GFP-transfected HeLa cells after incubation with the cis-platinum-loaded or blank eccentric magnetic microcapsules for different periods of time at two typical pH conditions.

Finally, we assessed the efficacy of cis-platinum released from the eccentric magnetic microcapsules in phantom tissues. In this study, the GFP-HeLa cells were encapsulated in gelatin hydrogels at varying pH conditions where the microcapsules (containing cis-platinum) were injected. After that, they were monitored under a fluorescence microscope. Fig. 7 shows the fluorescence images of the HeLa cells exposed to the cis-platinum-loaded or blank microcapsules for different periods of time at two typical pH conditions. In the experimental group, significantly fewer green cells were observed after 30 min (*p* < 0.01) and 90 min (*p* < 0.05) at pH 6.0 than at pH 7.0. The few deaths of cells in the control group (without cis-platinum) at pH 6.0 proved that it was the anti-cancer drug rather than the acidic surrounding that caused the cell deaths. These results also confirmed that our eccentric magnetic microcapsules were pH-responsive and the anti-cancer drug was released more quickly under a more acidic condition.

**Fig. 8:**
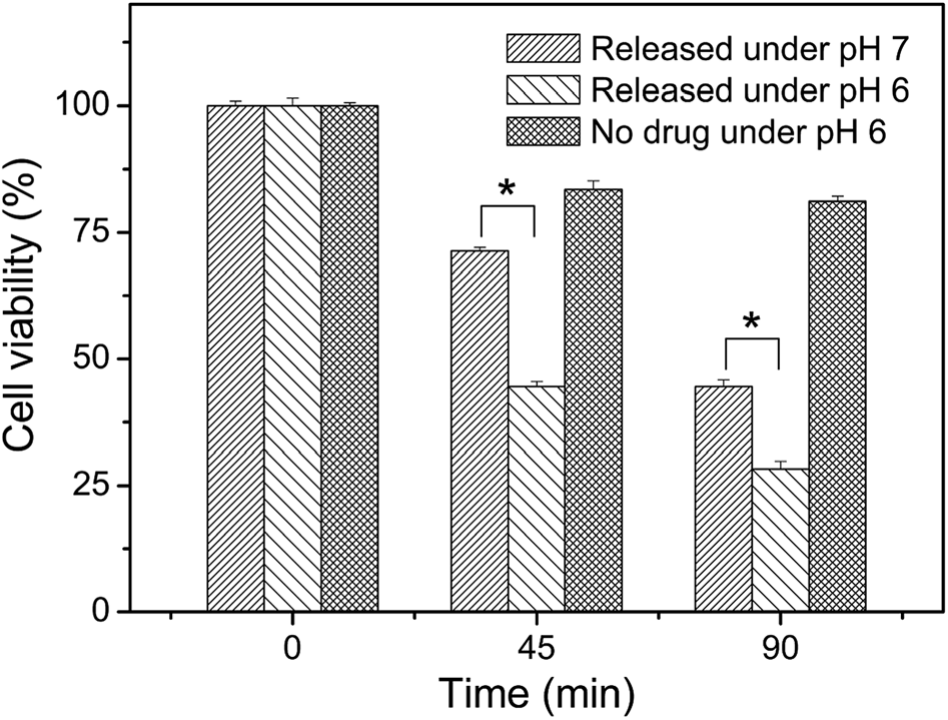
Viabilities of GFP-HeLa cells after incubation with the cis-platinum-loaded or blank eccentric magnetic microcapsules for different time intervals at two pH conditions. (*P < 0.05.)

Meanwhile, we also measured the viability of the fluorescent HeLa cells in Fig. 7 and the results were represented in Fig. 8. A time-dependent reduction of the fluorescent HeLa cells was found in each group. However, significantly more (*P* < 0.05) fluorescent HeLa cells were found in the experimental group at pH 7.0 than at pH 6.0, which confirmed once again that our magnetic microcapsules were pH-responsive and the release of the anti-cancer drug was faster at a more acidic condition. These results demonstrate that the drug release process from our eccentric magnetic microcapsules could still be regulated by pH even if they are injected into tissues.

## 4. Conclusions

In summary, we have developed a new class of uniform eccentric magnetic microcapsules for MRI-guided local administration and pH-controlled drug release. The magnetic nanoparticles are easy to be visualized by an MRI scanner, thereby achieving accurate imaging-guided local injection, and the release profiles and *in vitro* cell tests indicated that pH could regulate the release rates of drugs from the microcapsules. Compared with the microcapsules reported in previous literature, our drug delivery system based on the eccentric microcapsules containing magnetic nanoparticles offered a range of advantages: (i) we could achieve imaging-guided local injection using MRI or OCT; (ii) we could realize pH-regulated drug release as a consequence of the acidic dissolution of the embedded magnetic nanoparticles in the PDMS shell; and (iii) macromolecules or nanoparticles could be easily loaded and then released through the nanopores in the PDMS shells generated from the dissolution of the magnetic nanoparticles. We believe that such a novel microcarrier system, both visible in phantom tissues under MRI and OCT and pH-sensitive, holds great potential for diagnostics and therapeutics.

## Conflicts of interest

There are no conflicts to declare.

## Supplementary Material

RA-008-C8RA08501C-s001
