# Supplementary material for: Eccentric magnetic microcapsules for MRI-guided local administration and pH-regulated drug release
Source: RSC Adv. 2018 Dec 17;8(73):41956–65. doi: 10.1039/c8ra08501c (PMC9092120; doi:10.1039/c8ra08501c)
Supplement: RA-008-C8RA08501C-s001 [file RA-008-C8RA08501C-s001.pdf]

Electronic Supplementary Material (ESI) for RSC Advances.
This journal is © The Royal Society of Chemistry 2018

Supporting information for

Eccentric Magnetic Microcapsules for MRI-guided Local Administration and pH-regulated Drug Release

Jingxian Huang[a†], Wenwei Huang[a†], Yin Chen[a*], Yu Shrike Zhang[b], Jinshuang Zhong[a,c], Yan Li[a] and Jianhua Zhou[a*]

[a] J. Huang, W. Huang, Dr. Y. Chen, J. Zhong, Dr. Y. Li, Prof. J. Zhou
School of Biomedical Engineering, and The First Affiliated Hospital, Sun Yat-sen University,
Guangzhou 510006, China

[b] Prof. Y. S. Zhang
Division of Engineering in Medicine, Department of Medicine, Brigham and Women's Hospital, Harvard Medical School, 65 Landsdowne Street, Cambridge, Massachusetts 02139, United States

[c] Mr. J. Zhong
Imaging Department, Sun Yat-sen University Cancer Center, Sun Yat-sen University, Guangzhou 510080, China

*Corresponding authors. Tel.: +86 20 39387890; Fax: +86 20 39387890.
E-mail addresses:
chenyin8@mail.sysu.edu.cn (Y. Chen); zhoujh33@mail.sysu.edu.cn (J.H. Zhou).

†Huang, J. X. and Huang, W. W. contributed equally and are co-first authors.

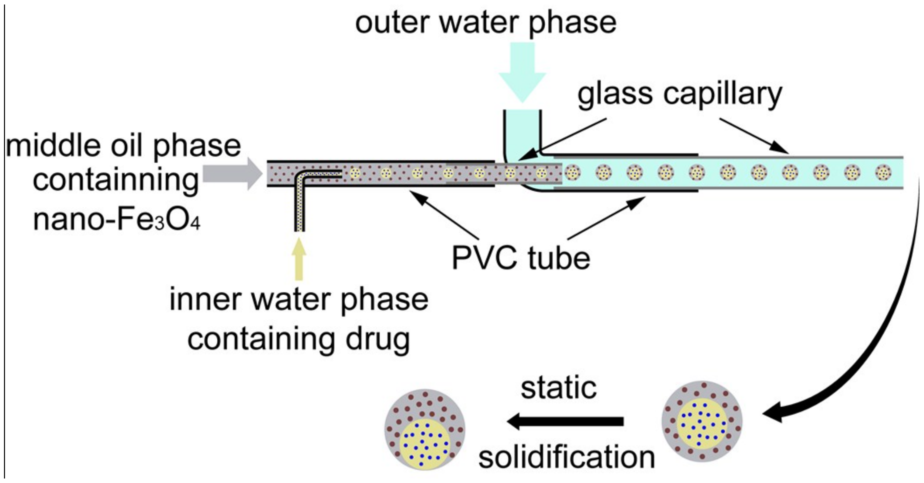

Figure S1. Generation of the pH-sensitive eccentric magnetic microcapsules by using a microfluidic device followed by stirring and baking.

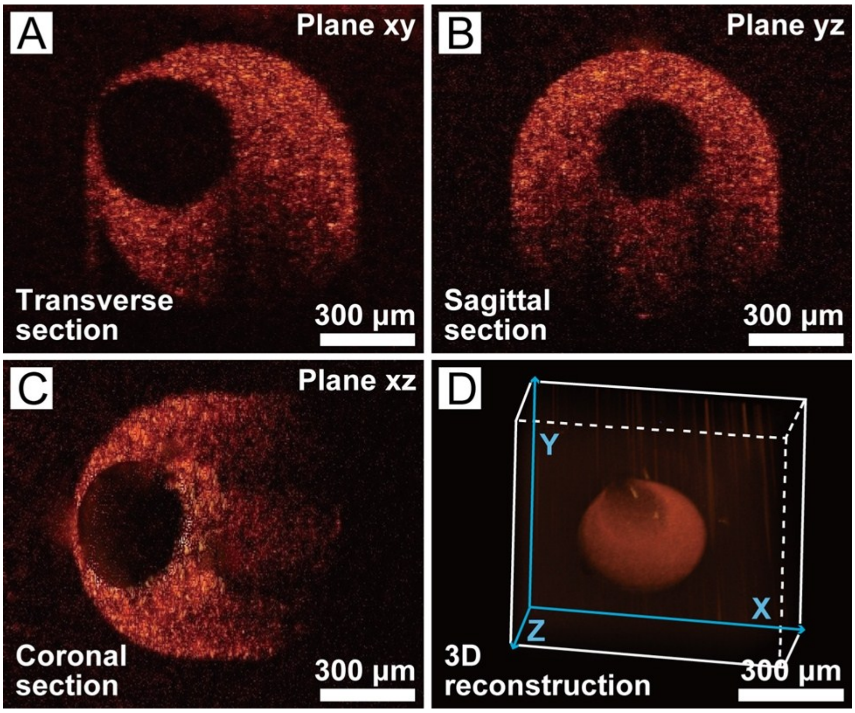

Figure S2. Optical coherence tomography (OCT) images of an eccentric magnetic microcapsule in a hydrogel showing the three-dimensional (3D) structure of it. (A) Image of transverse section. (B) Image of sagittal section. (C) Image of coronal section. (D) The 3D reconstructed image.

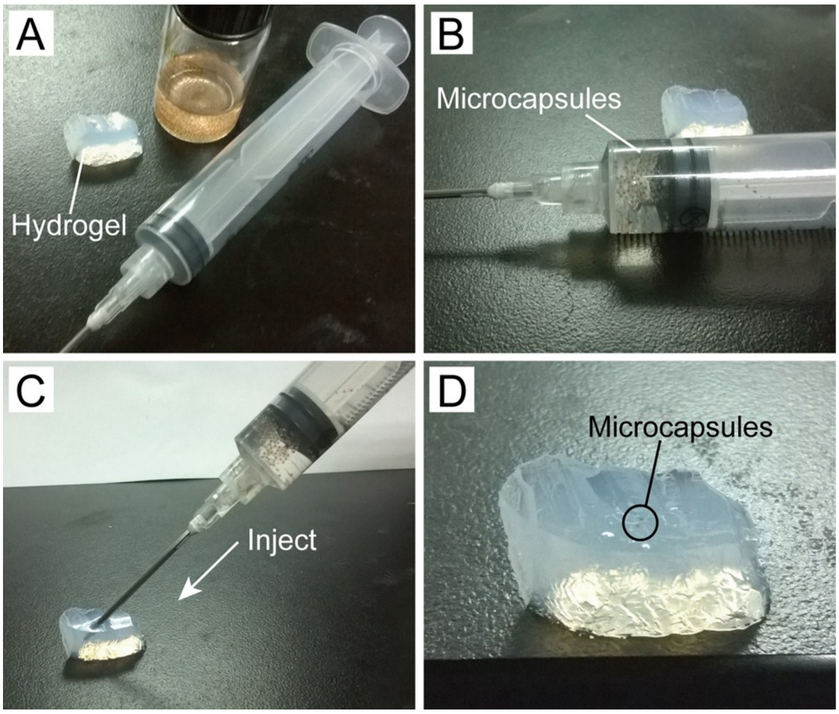

Figure S3. Local injection of the eccentric microcapsules into a hydrogel slab.

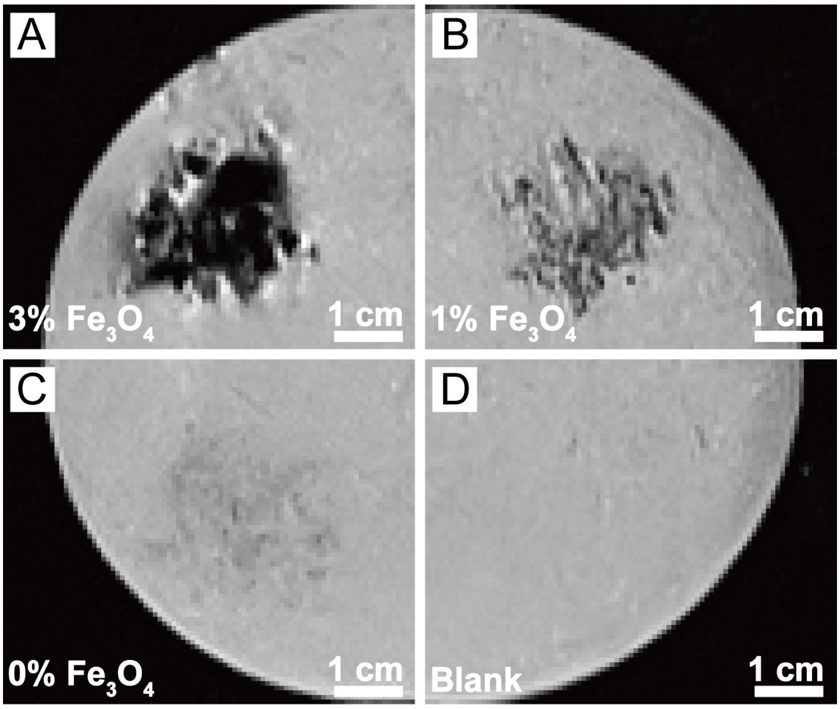

Figure S4. MRI images of the eccentric microcapsules with different mass fractions of the magnetic nanoparticles in a hydrogel slab. From (A) to (C), the mass fraction of the magnetic nanoparticles in the microcapsules decreased from 3.0% to 1.0%, and 0%. (D) MRI image of a blank hydrogel slab (without microcapsules).

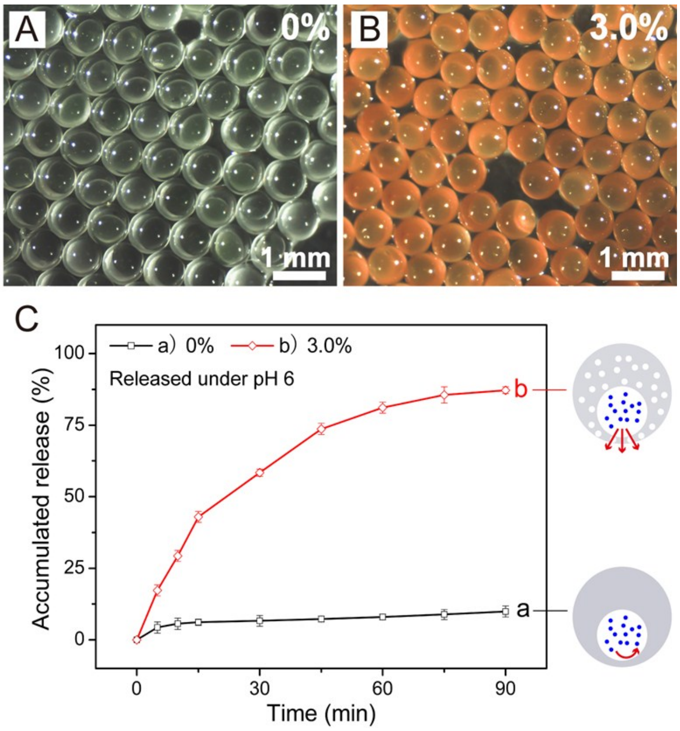

Figure S5. Comparison of the drug release profiles between the microcapsules embedded with and without the magnetic nanoparticles (Fe_3O_4). (A, B) Optical images showing the microcapsules without (A) or with 3.0% Fe_3O_4 (B). (C) The release rates of sodium fluorescein from the microcapsules without or with 3.0% Fe_3O_4 along with their respective release mechanisms (under pH 6).

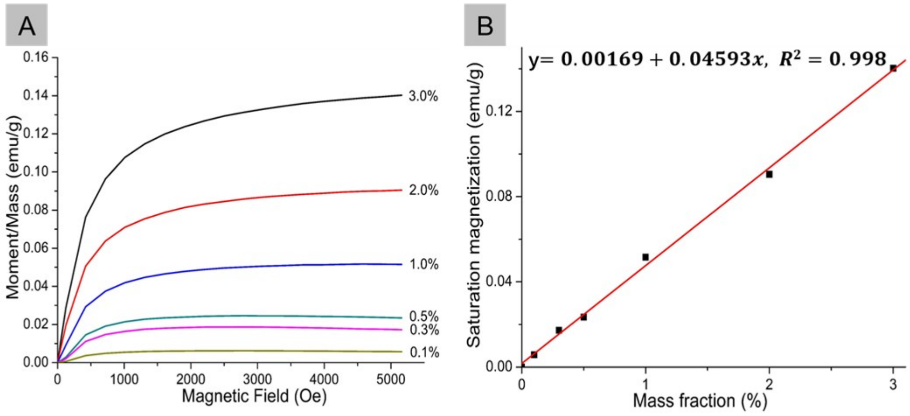

Figure S6. Specific saturation magnetization of the EMMs with different mass fractions of Fe_3O_4 nanoparticles. (A) Change in specific saturation magnetization for the EMMs containing 0.1%, 0.3%, 0.5%, 1.0%, 2.0%, and 3.0% Fe_3O_4 nanoparticles, respectively. (B) The linear fitting for the correlation between specific saturation magnetization and mass fraction of Fe_3O_4 nanoparticles.

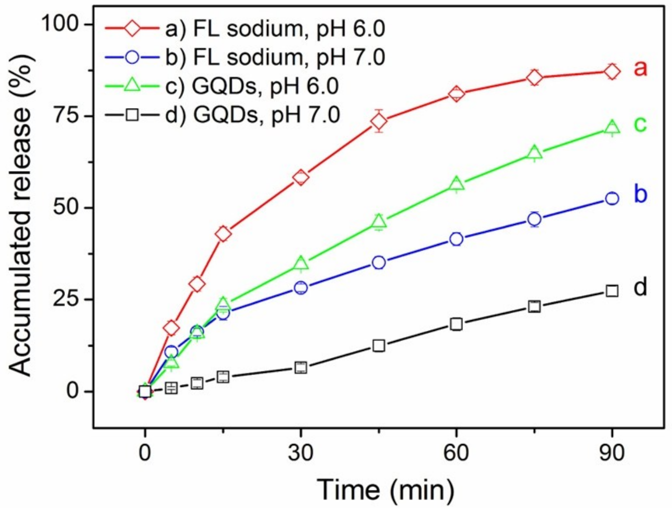

Figure S7. Comparison of the release profiles between GQDs and sodium fluorescein under pH 6 and 7.

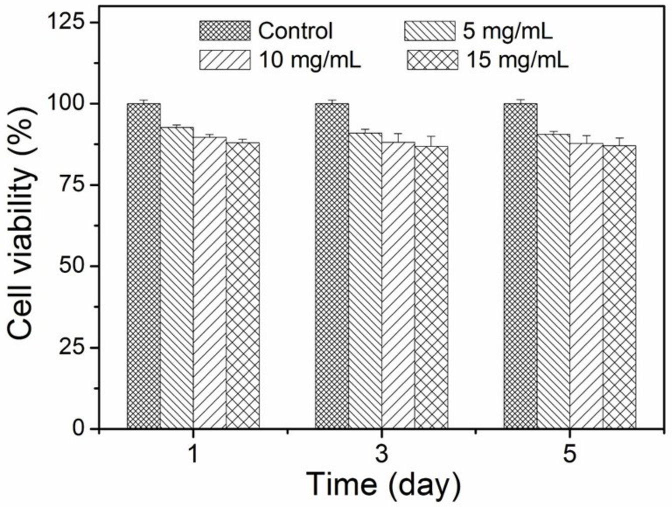

Figure S8. Viabilities of HeLa cells treated with different concentrations of the microcapsules for different days evaluated by MTT assay. The cell viabilities were still higher than 80% even after incubation with high concentrations of the microcapsules for 5 days. (P>0.1)

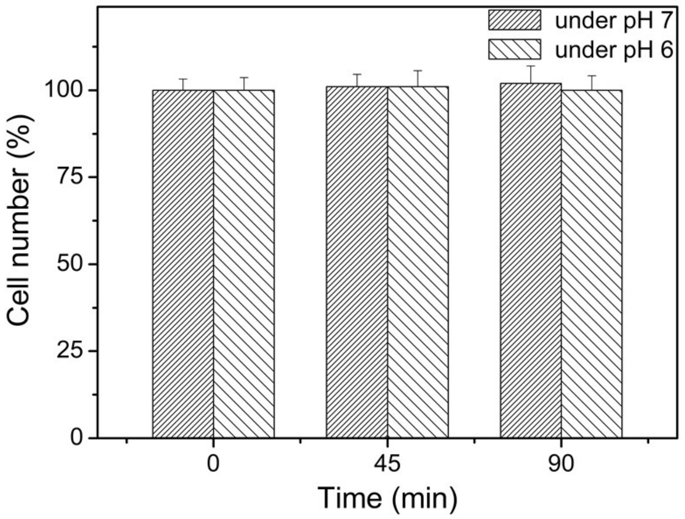

Figure S9. Viabilities of GFP-HeLa cells after incubation at two pH conditions for different time intervals.
